# Cerebral amyloid angiopathy aggravates perivascular clearance impairment in an Alzheimer’s disease mouse model

**DOI:** 10.1186/s40478-020-01042-0

**Published:** 2020-11-05

**Authors:** Shin Heun Kim, Ji Hoon Ahn, Hyunwoo Yang, Peter Lee, Gou Young Koh, Yong Jeong

**Affiliations:** 1 Program of Brain and Cognitive Engineering, Korea Advanced Institute of Science and Technology (KAIST), Daejeon, Republic of Korea; 2 Department of Bio and Brain Engineering, KAIST, Daejeon, Republic of Korea; 3 KI for Health Science and Technology, KAIST, Daejeon, Republic of Korea; 4 Graduate School of Medical Science and Engineering, KAIST, Daejeon, Republic of Korea

**Keywords:** Cerebral amyloid angiopathy, Alzheimer’s disease, Intramural periarterial drainage, Perivascular cerebrospinal fluid influx

## Abstract

Cerebral amyloid angiopathy (CAA), defined as the accumulation of amyloid-beta (Aβ) on the vascular wall, is a major pathology of Alzheimer’s disease (AD) and has been thought to be caused by the failure of Aβ clearance. Although two types of perivascular clearance mechanisms, intramural periarterial drainage (IPAD) and the perivascular cerebrospinal fluid (CSF) influx, have been identified, the exact contribution of CAA on perivascular clearance is still not well understood. In this study, we investigated the effect of CAA on the structure and function of perivascular clearance systems in the APP/PS1 transgenic mouse model. To investigate the pathological changes accompanied by CAA progression, the key elements of perivascular clearance such as the perivascular basement membrane, vascular smooth muscle cells (vSMCs), and vascular pulsation were evaluated in middle-aged (7–9 months) and old-aged (19–21 months) mice using in vivo imaging and immunofluorescence staining. Changes in IPAD and perivascular CSF influx were identified by ex vivo fluorescence imaging after dextran injection into the parenchyma or cisterna magna. Amyloid deposition on the vascular wall disrupted the integrity and morphology of the arterial basement membrane. With CAA progression, vascular pulsation was augmented, and conversely, vSMC coverage was decreased. These pathological changes were more pronounced in the surface arteries with earlier amyloid accumulation than in penetrating arteries. IPAD and perivascular CSF influx were impaired in the middle-aged APP/PS1 mice and further aggravated in old age, showing severely impaired tracer influx and efflux patterns. Reduced clearance was also observed in old wild-type mice without changing the tracer distribution pattern in the influx and efflux pathway. These findings suggest that CAA is not merely a consequence of perivascular clearance impairment, but rather a contributor to this process, causing changes in arterial function and structure and increasing AD severity.

**Electronic supplementary material:**

The online version of this article (10.1186/s40478-020-01042-0) contains supplementary material, which is available to authorized users.

## Introduction

Alzheimer’s disease (AD) is the most common type of dementia, characterized by the accumulation of amyloid plaques and hyperphosphorylated tau. Recent studies have suggested that excessive amyloid plaques may be the result of an imbalance between production and clearance rather than overproduction of amyloid-beta (Aβ) [31, 40]. Growing evidence supports that the removal rate of Aβ is impaired in both early-onset and late-onset forms of AD; thus there is an increasing demand to investigate the Aβ clearance mechanism as a potential target for AD treatment.

Aβ is known to be removed from the brain in different ways, such as degradation by proteases, transportation through the blood–brain barrier, and removal by the flow of interstitial fluid (ISF) and cerebrospinal fluid (CSF) [23, 45]. According to recent studies, there are two types of perivascular clearance systems, one related to the movement of ISF and the other to CSF, which are called intramural periarterial drainage (IPAD) and perivascular CSF influx, respectively [6, 45].

IPAD is the perivascular clearance process where parenchymal ISF containing Aβ exits the brain along the basement membrane (BM) of the capillary and arterial smooth muscle cells, and finally drains into the cervical lymph nodes [6, 10, 43]. Cerebral amyloid angiopathy (CAA), in which Aβ mainly accumulates in the BM of capillary and arterial smooth muscle cells, is thought to be the result of IPAD failure, since the accumulation pattern is consistent with the IPAD pathway [5, 10, 47]. In addition, augmented CAA in AD patients receiving immunotherapy supports the view that IPAD is the major pathway for Aβ elimination, as dissolved Aβ generated by the treatment could exacerbate the existing CAA in the process of its removal through IPAD [11, 15, 39].

CSF moves in synchronization with vascular pulsation along the pial-glial BM [21, 28, 29]. The astrocyte end-feet that form the outermost boundary around vessels, called glia limitans, express the water channel aquaporin 4 (AQP4), which mediates the influx of CSF within the brain parenchyma. [20, 22]. Depletion of AQP4 in the AD transgenic mouse model has been observed and causes accelerated amyloid accumulation and aggravated cognitive deficit, and these findings suggest that the perivascular CSF inclux is also involved in Aβ removal [41, 49].

In particular, the artery has perivascular compartments consisting of several layers of BM including the BMs of endothelial cells, vSMCs, and those of glia limitans and pia mater. The CSF influx is occurring in the potential space between the glia limitans and the BM of vSMCs, while IPAD occurs along the BM of the vSMCs. BM is a major component of the perivascular clearance pathway for soluble proteins and fluids to enter or exit the brain [3, 17, 35], so it is important to investigate the how amyloid deposition alter perivascular compartments.

Furthermore, vascular pulsation is considered as the driving force of both systems, despite the fact that they are thought to flow in opposite directions around the periarterial side. Vascular pulsation is a kind of vascular movement and is defined as an alternating increase and decrease in vessel diameter driven mainly by the heartbeat. CSF movement around the arteries is influenced by the vascular pulsation [21, 28, 29]. The IPAD is also known to be associated with vascular pulsation based on the findings that increased heart rate facilitates IPAD by promoting solute distribution within brain parenchyma [16, 40]. Vascular pulsation contributes to both systems by inducing subsequent CSF and ISF movements in the perivascular compartments and extracellular space. However, little is known about the changes in these key factors in perivascular clearance systems in pathological conditions such as AD.

In this study, we investigate how CAA alters the structure and function of vascular components, which are considered key factors in perivascular clearance, and ultimately influences the perivascular clearance. Using in vivo and ex vivo imaging and histological approaches, we demonstrated that progressive pathological changes including morphological alteration in the periarterial BM, loss of vSMCs, and augmented vascular pulsation are accompanied by CAA progression. From the early stages of CAA, these pathological changes and clearance impairment are detected; the former becomes more pronounced and the latter worsens with CAA progression. Moreover, the perivascular CSF influx and solutes efflux patterns were severely disrupted in AD transgenic mice. These findings indicate that CAA leads to vascular pathological changes and ultimately aggravates Aβ clearance mechanisms.

## Materials and methods

### Animals and surgery

APPswe/PSEN1dE9 (APP/PS1) mice with a B6C3 hybrid background were purchased from Jackson Laboratory (USA, #004462). Transgenic mice (Tg) were crossed with wild-type B6C3 F1 mice (Wt) to maintain hemizygotes. Both male and female Tg and age-matched Wt littermates at 7- to 9-months-old (mid) and 19- to 21-months-old (old) were used. A total of 96 mice were used in this study. Each experiment included at least two females, and sex balance was maintained at approximately 50%: 50% in all experiments (Additional file 1). In APP/PS1, CAA is known to initiate after about 6 months [26], so two age groups were chosen to confirm the changes in CAA progression. All mice were housed under a 12 h light/dark cycle. Food and water were freely accessible. All procedures were approved by the KAIST Institutional Animal Care and Use Committee. Animal care and handling were performed in accordance with their guidelines.

For in vivo imaging, animals were anesthetized by intraperitoneal injection of urethane (1.5 g/kg). To prevent secretion, glycopyrrolate (0.02 mg/kg) was injected subcutaneously, and a tracheotomy was performed to prevent trachea blockage by phlegm. The center of the cranial window (3 mm in diameter) was 2.5 mm posterior from bregma and 2.5 mm laterally from the midline. A custom head plate was secured to the skull using dental cement. The cranial window was covered with a 5 mm diameter cover glass soaked with 1.5% agarose to reduce movement artifacts during the imaging procedure. To avoid agarose shrinkage during the experiment, dental cement wells were filled with artificial CSF (aCSF) and then covered with 12 mm cover glass. During the surgical and imaging process, the body temperature of the mouse was maintained at 37 °C using a heating pad (TCAT-2LV, physitemp) with a feedback control system.

### In vivo two-photon microscopy imaging

To visualize vasculature, 2 MDa FITC-dextran (50 mg/kg; Thermo Fisher Scientific) was injected via the tail vein. To tag amyloid plaques, methoxy-X04 (MX04, 5 mg/kg) was administered intraperitoneally 1 day before. The cortex was imaged using a two-photon microscope (Nikon A1 plus, Nikon Instruments) and a 25 × water immersion objective (NA 1.1, Nikon) was used throughout the experiment. The excitation wavelengths were 850 nm for vasculature and 800 nm for amyloid plaque. Angiography images were acquired at 512 × 512 pixels with 5-µm z-steps, and vascular pulsation was measured using a line scan. Vessels located within 150 µm of the cortical surface were selected for pulsatility index measurement. The scanning lines for the pulsatility index measurement were set orthogonal to the path of blood vessels. The scanning lines for penetrating arteries and ascending veins were chosen where the vascular cross-section appeared circular and located 50-150 µm below the surface. Body temperature was maintained at 37 °C through a feedback-controlled heating pad during the entire imaging session.

### Vascular pulsation analysis

The method described in a previous study was used to quantify the vascular pulsation [21]. Line scan data were extracted and plotted against time using MATLAB software with customized code. The vascular wall pulsatility index (derived unit [µm × ms]) was calculated by integration of the difference between the diameter curve and average curve over 3000-ms epochs. The average vessel diameter was calculated by the moving average function.

$$ \begin{aligned} & {\textit{Pulsatility index}} = \int_{0}^{3000} {\left| {D\left( t \right) - d\left( t \right)} \right|}    \\ & D\left( t \right) = {\textit{vessel diameter}},\quad d\left( t \right) = {\textit{moving average of vessel diameter}} \\ & {\textit{Relative pulsatility index}} = \int_{0}^{3000} {\left| {\frac{D\left( t \right) - d\left( t \right)}{{{\textit{mean}} \left( {D\left( t \right)} \right)}}} \right|}    \\ \end{aligned} $$

The pulsatility index was calculated by two methods. The pulsatility index measured the absolute movement and the relative pulsatility index measured the relative movement with respect to the size of the blood vessel. The relative vascular wall pulsatility index was calculated by dividing the absolute difference between the diameter curve and average curve by the vessel diameter and then integrated over the 3000-ms epochs. The vessel diameter was used as the static average diameter during the line scan image. The smoothing function was used to exclude excessive high-frequency information. All data were analyzed in MATLAB with custom code.

### Cisterna magna injection

Three kDa fluorescein isothiocyanate (FITC)-labelled dextran (3 k-FITC, D3305, Invitrogen) and 40 kDa tetramethylrhodamine (TMR)-labelled dextran (40 k-TMR, D1842, Invitrogen) was dissolved in aCSF and delivered through cisterna magna (CM). 3 k-FITC has a molecular weight similar to that of the Aβ monomer and is known to have access to the brain independent of AQP4. 40 k-TMR is suitable to examine the perivascular CSF influx affected by AQP4. During the experiment, the heart rate was monitored with a set of three platinum needle electrodes connected to the data acquisition system iX228s (Iworx). The head was fixed to the stereotactic frame, and the head and neck were positioned at 120°. After incising the dorsal neck skin, the neck muscles were exposed with a retractor to access the atlanto-occipital membrane overlying the CM. A disposable 30 G needle was carefully inserted and fixed with a cyanoacrylate bond while avoiding contact with the medulla or cerebellum. The solution was delivered by an automatic syringe pump (KD Scientific) for 5 min at 2 µl/min and cardiac perfusion was performed 30 min after the injection [48].

### Intraparenchymal injection

A solution of 3 k-FITC dissolved in aCSF at 0.1% concentration was injected into the brain parenchyma. The solution was loaded in a glass capillary with a diameter of less than 60 µm connected to an automatic syringe pump (KD Scientific) to deliver the solution at 0.1 µl/min for 5 min. The injection site was the left somatosensory cortex (AP: − 0.5 mm, ML: − 2.2 mm, DV: − 1.3 mm). To prevent reflux, the glass capillary was left for 10 min after injection, and cardiac perfusion was performed 30 min after intra parenchymal injection.

### Immunofluorescence

Mice were perfused with ice-cold phosphate-buffered saline (PBS) followed by 4% paraformaldehyde (PFA) in PBS. The extracted brain was fixed with 4% PFA for 4 h at 4 °C. Samples were cut into 150-µm thick coronal sections using a vibrating microtome (Leica) and post-fixed with 4% PFA for 30 min at 4 °C. For immunofluorescence staining, samples were permeabilized and blocked for 1 h at room temperature with a blocking buffer containing 5% donkey or goat serum in 1% Triton X-100/1 × PBS. Samples were then incubated overnight at 4 °C with the following primary antibodies: anti-αSMA–FITC (mouse monoclonal, F3777, Sigma-Aldrich, 1:400) for vascular smooth muscle cells; anti-collagen IV (rabbit polyclonal, ab6586, Abcam, 1:400) for the basement membrane; and anti-GFAP (chicken polyclonal, ab4674, Abcam, 1:400) for astrocytes. After washing several times with PBS, samples were incubated overnight at 4 °C with Alexa Fluor-conjugated secondary antibodies (Alexa 488-, 647-conjugated, Jackson ImmunoResearch). After washing several times with PBS, the samples were mounted with mounting medium (Vectashield, Vector Laboratories).

### Immunofluorescence data analysis

Immunofluorescence images were acquired with a Plan-Apochromat 20×/0.8 NA M27 confocal microscope (LSM800, Carl Zeiss). The confocal images were taken in a tiled z-stack with 5-µm steps and then merged into a single image by the full intensity projection function of Zen 2.3 software (Carl Zeiss). The region of interest (ROI) of each blood vessel was defined through the selection of a freehand line function in Fiji, and the surface artery or the penetrating artery was manually identified.

Seven to twelve brain slices were acquired from each mouse to quantify the coverage of amyloid deposition and vSMCs in the surface and penetrating arteries. Thirteen to forty-two vessels per mouse were measured and 4 animals per group were used. After merging the images of the SMA and MX04 channels into one, the threshold was applied to create masks for each vessel. The coverage was determined by calculating the percentage of positive pixels present in the vascular mask in the image of each channel. The vascular density was measured as the percentage occupied by the collagen IV-tagged vessels in a constant sized ROI of 0.4 mm × 0.4 mm. Two slices per mouse were used for measuring the vascular density. All the analyses mentioned above were performed using the Fiji software.

### Ex vivo fluorescence imaging

The brains injected with tracer through the CM were immersed in 4% PFA overnight and cut into coronal sections 100-µm thick using a vibratome (Leica). The brains injected with tracer into the parenchyma were fixated in 4% PFA for 4 h at 4 °C, and then cut into slices of 100-µm thickness. The sliced brains were further fixed for 30 min in 4% PFA, and then the sample were blocked at room temperature for 1 h with a blocking solution containing 5% goat serum in 1% Triton X-100/1 × PBS. Samples were incubated overnight at 4 °C with anti-αSMA–CY3 (mouse monoclonal, C6198, Sigma-Aldrich, 1:400) to label vascular smooth muscle cells. Samples were mounted with mounting medium (Vectashield, Vector Laboratories) and imaged using a whole-slide imaging system (10 × objective; Axio Scan Z1, Zeiss) to analyze brain parenchyma and perivascular tracer signals. To closely observe the distribution of dextran in detail in the perivascular compartments and parenchyma, some vessels were further imaged using a confocal microscope (LSM 880, Carl Zeiss). Images were acquired with z-stacks in 5-µm steps and then converted to 2D images with maximum projection. Zen software (Carl Zeiss) was used for acquisition and image processing.

### Ex vivo fluorescence signal analysis

To quantify IPAD, a total of 18 brain slices were obtained from each mouse, up to 0.9 mm anterior and posterior to the injection site. The number of blood vessels with both SMA and tracer signals was manually counted. Only the tracer injected hemisphere was used for the analysis. In the CM injection experiment, a total of 7 slices were obtained from all mice, in one slice every 5 slices, from 1 mm anterior from the bregma to 2 mm posterior to the bregma for analysis of the whole-brain fluorescence signal. To measure the total brain influx signal, the brain slice was set as one ROI and the background-subtracted procedure was performed. Brain images for total brain influx signal analysis were also used to count the number of tracer positive penetrating vessels. Fluorescent images of the two tracers were merged and the number of penetrating vessels perpendicular to the cortex were counted. The cortex was divided into lateral, dorsal, and ventral parts to measure the regional differences between the fluorescent signals in the brain. The dorsal, lateral, and ventral regions were distinguished by the primary somatosensory cortex and the piriform cortex. The brain images were manually registered to the template, and signals from each part were analyzed. To quantify amyloid plaques, a constant threshold was applied to the background-subtracted image, and the amyloid burden in each region was calculated as the ratio of plaque in the regional mask. The parenchymal plaque coverage, defined as (plaque coverage = thresholded pixel in MX04 channel/brain ROI size × 100), was calculated. All procedures were performed using Fiji.

### Statistical analysis

All data are shown as the mean ± standard error of the mean (SEM). Two-way analysis of variance (ANOVA) with Bonferroni’s post hoc comparison was used to evaluate the effect of genotype and age and identify differences within groups. One-way ANOVA with Tukey’s post hoc comparison was used to assess the regional differences in amyloid burden and fluorescence signal. Linear regression was performed to investigate the relationship between amyloid burden and CSF influx signal. GraphPad Prism 8 software was used for the statistical analysis. In all cases, *p* < 0.05 was considered statistically significant.

## Results

### CAA disrupts the morphology and integrity of the periarterial basement membrane

**Fig. 1:**
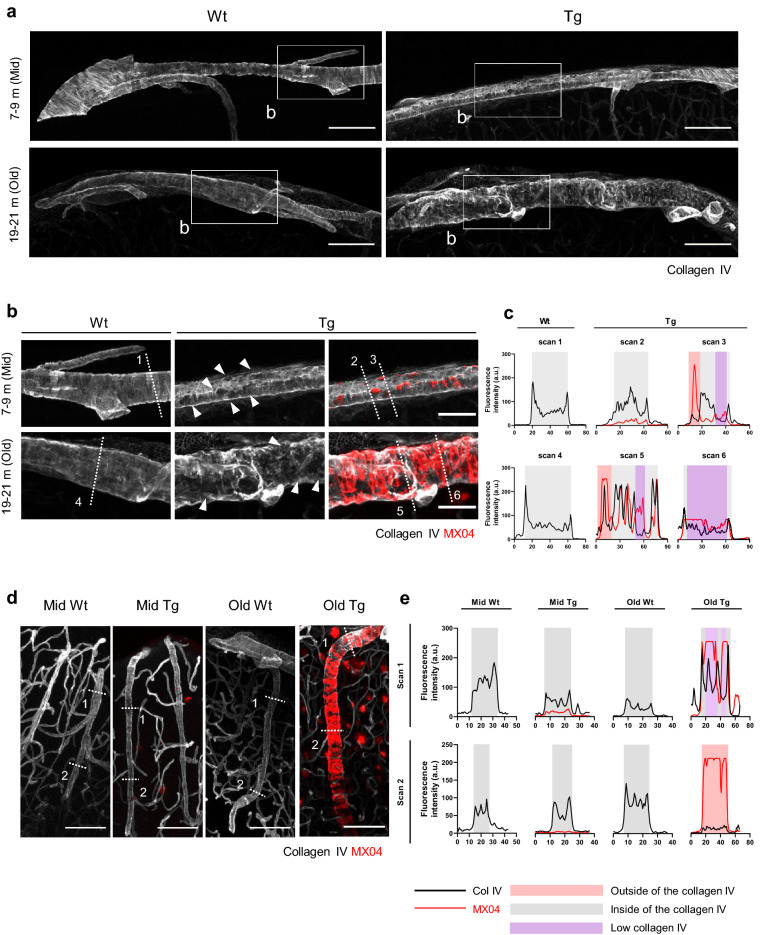
Disruption of the morphology and integrity of periarterial basement membrane in cerebral amyloid angiopathy. **a** Morphology of the periarterial basement membrane (BM) in the surface arteries. Collagen IV was used to identify the BM. Note the discontinuity in the morphology of collagen IV in mid and old Tg. The inlet boxes indicate the areas with higher magnification images in (**b**). Scale bar = 100 µm. **b** Representative images of the BM (collagen IV) and amyloid plaque (MX04) in the surface arteries. The collagen IV layer with amyloid plaque deposition is pierced (white arrowhead). Note that the collagen IV layer appears distorted in accordance with the shape of amyloid accumulation in old Tg. The white dotted lines represent the scanning lines for intensity profile analysis in (**c**). Scale bar = 50 µm. **c** Intensity profile analysis of the fluorescence signal of collagen IV and MX04 in the surface arteries. The numbers indicate each scan line in (**b**). Note that the MX04 signal is mainly observed within the collagen IV layer (gray-shaded area) but also outside the layer in old Tg (red-shaded area). The collagen IV intensity decreased with an increase in the MX04 signal (purple-shaded area). **d** Representative images of the BM (collagen IV) and amyloid plaque (MX04) in the penetrating arteries. The white dotted lines represent the scan lines for intensity profile analysis in (**e**). Scale bar = 100 µm. **e** Fluorescence intensity profile of collagen IV and MX04 in the penetrating arteries. The numbers indicate scan lines in (**d**). Scan 1 depicts the superficial part, while scan 2 depicts the deep cortical part

We examined whether the amyloid deposits cause pathological changes in the BM of surface and penetrating arteries. BM consists of collagen IV, laminins, nidogens, and heparin sulfate proteoglycans, and collagen IV was used as a marker for periarterial BM [34]. In the surface arteries, the collagen IV signal showed a striped pattern similar to the arrangement of vSMCs in Wt (Fig. 1a). In the mid Tg, there was no apparent change in the overall BM morphology, but the collagen IV layer was perforated in the presence of plaques (Fig. 1b, white arrowhead). In the old Tg, the morphology of the collagen IV layer was significantly distorted corresponding to the shape of amyloid deposition (Fig. 1b). Collagen IV expression generally shows two peaks at the borders of the BM in Wt; however, a hole was observed in the artery segments with amyloid deposits and collagen IV signal in this region was reduced (Fig. 1c, purple rectangle) in Tg. Similar to the previous results, the amyloid deposition was mainly observed underneath the collagen IV layer (Fig. 1c, gray rectangle) but amyloid deposits were also detected outside of the collagen IV layer in the area with severe BM disruption (Fig. 1c, red rectangle).

The spatial distribution of collagen IV in the penetrating arteries also has a clear boundary, which was severely damaged in old Tg. Especially, there was a difference between the superficial and deep parts (Fig. 1d, e). On the deep part, the boundary of the collagen IV signal disappeared more, and more amyloid plaques accumulated outside of the BM (Fig. 1e, red rectangle), whereas, the BM located on the surface side was relatively well preserved and the amyloid deposition accumulated inside of the BM (Fig. 1e, gray rectangle) in old Tg. Together, vascular Aβ deposition results in morphological change and integrity disruption of the BM, indicating amyloid plaques could accumulate inside and outside of the periarterial BM. These changes were more evident in old Tg with advanced CAA.

### The progression of CAA leads to loss of vascular smooth muscle cells and augmented vascular pulsation

vSMCs are contractile mural cells that enclose the wall of arteries and directly control the blood vessel diameter [19]. The coverage of vSMCs around blood vessels is an important indicator for vascular movement; therefore, we examined the changes of vSMCs and vascular movement in relation to CAA deposition.

**Fig. 2:**
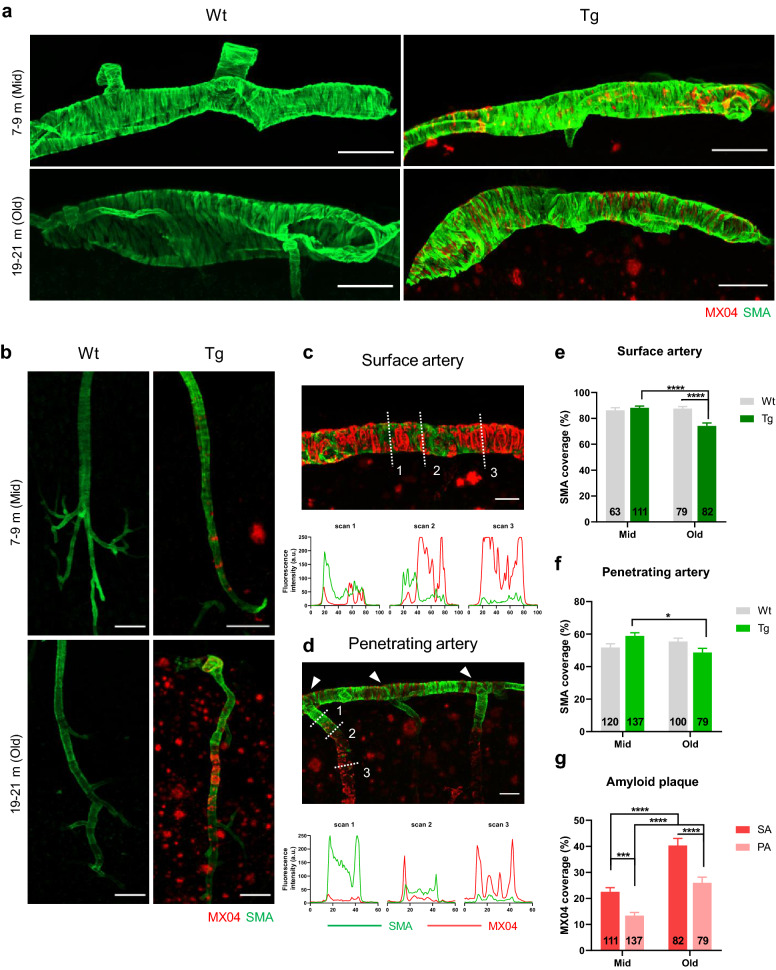
Loss of vascular smooth muscle cells due to the progression of cerebral amyloid angiopathy. **a, b** Representative images of the vascular smooth muscle cells [vSMCs; smooth muscle actin (SMA)] and amyloid plaque (MX04) in the surface arteries (**a**) and penetrating arteries (**b**). Note the Aβ plaque accumulation between the vSMCs in the surface arteries, which completely envelops the blood vessels in the penetrating arteries. Scale bar = 100 µm. **c, d** Comparison of the distribution of SMA and MX04 in the surface arteries (**c**) and penetrating arteries (**d**) in old Tg. The white dotted lines represent the scan lines for intensity profile analysis. Note that the SMA and MX04 signals exhibit complementary profiles in both types of arteries. Scale bar = 50 µm. **e, f** Quantification of the coverage of vSMCs in the surface (**e**) and penetrating arteries (**f**). [**e**. surface arteries; *p* < 0.0001 (genotype*age interaction), *p* = 0.0005 (age), *p* = 0.0016 (genotype)], [**f**. penetrating arteries; *p* = 0.00211 (genotype*age interaction)]. **g** Quantification of the coverage of amyloid plaque in the surface and penetrating arteries [*p* < 0.0001 (age), *p* < 0.0001 (vessel type)]. 13 to 42 vessels per mouse and n = 4 per group. All data are presented as the mean ± SEM. **p < 0.01, ***p < 0.001, ****p < 0.0001. Two-way ANOVA with Bonferroni’s post hoc test

To illustrate the relationship between vSMCs and amyloid accumulation, each was identified by smooth muscle actin (SMA) and MX04, respectively. In the surface arteries, amyloid (MX04) accumulated between the vSMCs (SMA) surrounding the arteries like a ring, whereas, in the penetrating arteries, Aβ completely covered the outer wall of the blood vessels (Fig. 2a, b). In both surface and penetrating arteries, SMA and MX04 signals showed opposite intensity profiles, indicating that areas with high amyloid accumulation showed reduced vSMCs coverage (Fig. 2c, d). Also, the loss of the SMA signal in the penetrating artery was more pronounced in the deep part than the superficial part (Fig. 2d). In particular, vSMCs of the transition zone, where the surface arteries become penetrating arteries, were relatively well preserved against amyloid accumulation compared to the penetrating artery located in the deep cortex (Fig. 2d, white arrowhead).

We quantified the coverage of vSMCs and amyloid deposition in surface and penetrating arteries (Fig. 2e–g). vSMCs were relatively preserved in mid Tg but significantly disappeared in surface arteries in old Tg compared with mid Tg and old Wt (Fig. 2e) (mid Tg vs. old Tg, *p *< 0.0001; old Wt vs. old Tg, *p *< 0.0001; two-way ANOVA with Bonferroni’s post hoc comparison). The vSMC coverage of penetrating arteries decreased in old Tg compared to mid Tg (Fig. 2f) (mid Tg vs. old Tg, *p* = 0.0104). The amount of amyloid deposition differed across the cerebrovascular tree and was greater in the surface arteries than penetrating arteries (Fig. 2g) (mid SA vs. mid PA, *p* = 0.0006; old SA vs. old PA, *p* < 0.0001). In the surface arteries, the amyloid deposition was clearly observed in mid and old Tg (Additional file 2, white arrowhead), while in the penetrating arteries, the amyloid deposits became apparent in old Tg (Additional file 2, yellow arrowhead). Moreover, the amyloid deposition was greater in the penetrating arteries with larger diameters (Additional file 2, yellow arrowhead).

**Fig. 3:**
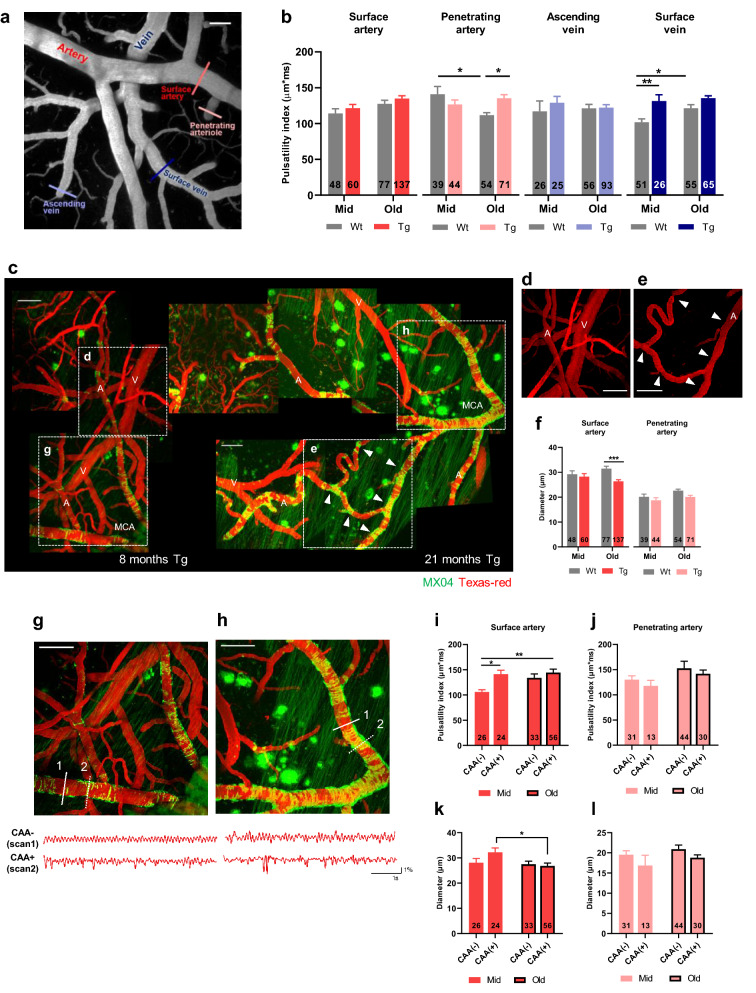
Augmentation of vascular pulsation with cerebral amyloid angiopathy progression. **a** Representative in vivo image of the vasculature obtained using two-photon microscopy. Vascular pulsations were recorded using line scanning, and measured along the cerebrovascular tree. Each vessel type in the cerebrovascular tree is marked with a different color. Cortical surface artery (red line), penetrating artery (light red line), ascending vein (light blue line), and surface vein (navy line). Scale bar = 50 µm. **b** Pulsatility index in the cerebrovascular network. [surface artery, *p* = 0.0132 (age); penetrating artery, *p* = 0.0032 (genotype*age interaction); surface vein, *p* = 0.0253 (age), *p* < 0.0001 (genotype), two-way ANOVA) (mid Wt = 6, mid Tg = 6, old Wt = 6, old Tg = 7)]. **c** Progression of CAA in APP/PS1 mice. Amyloid accumulation in the middle cerebral artery (MCA) can be observed in mid (left) and old Tg (right). CAA is not observed in the veins (V). Scale bar = 100 µm. **d, e** Magnified image of the surface artery. The arterial diameter (A) remains relatively constant compared to the venous diameter (V) in mid Tg (**d**). Note the reduction in arterial diameter (white arrowhead) in segments with excessive CAA in old Tg (**e**). Scale bar = 100 µm. **f** Comparison of arterial vascular diameter. The decrease in vascular diameter was significant in surface arteries. [surface artery, *p* = 0.0374 (genotype*age interaction), *p* = 0.0028 (genotype); penetrating artery, *p* = 0.0219 (age), *p* = 0.0186 (genotype)]. **g, h** Comparison between vascular pulsation in segments with (dotted white lines) and without CAA (white lines) in mid (**g**) and old Tg (**h**). Scale bar = 100 µm. **i, j** Effect of CAA on vascular pulsation in surface (**i**) and penetrating arteries (**j**). [surface artery, *p* = 0.0037 (CAA), *p* = 0.0449 (age)]. Note that the pulsatility index markedly increased in the CAA-positive vessels in mid Tg (mid Tg; n = 6, old Tg; n = 7). **k, l** Effect of CAA on the vascular diameter of surface (**k**) and penetrating arteries (**l**). [surface artery, *p* = 0.0430 (age)]. Note that the decrease in vascular diameter is more prominent in CAA-positive vessels. All data are presented as the mean ± SEM. *p < 0.05, **p < 0.01, ***p < 0.001, ****p < 0.0001 two-way ANOVA with Bonferroni’s post hoc test

Vascular pulsation was measured along cerebrovascular trees by line scan imaging (Fig. 3a). Since the continuous movement of blood vessels induces the flow of fluid around the artery and adjacent extracellular space, vascular pulsation has been considered as a driver for perivascular clearance [16, 21]. In order to avoid the effects of vascular diameter change caused by CAA on the pulsatility index, the index was calculated as an absolute extent of the blood vessel movement without including diameter information. The pulsatility index values of surface arteries, penetrating arteries, and surface veins were the highest in old Tg (Fig. 3b). Specifically, the pulsatility index of penetrating arteries increased in old Tg compared to old Wt (old Wt vs. old Tg, *p* = 0.0216), and decreased in old Wt compared with mid Wt (mid Wt vs. old Wt, *p* = 0.0123).

Amyloid deposition was mostly observed on the arterial side but was rarely found on the venous side (Fig. 3c). Angiography images show that blood vessels with a high amyloid burden contracted in old Tg, and this change was not observed in mid Tg (Fig. 3d, e, white arrow). In terms of vascular diameter, surface arteries of old Tg had lower values than those of mid Tg and old Wt (Fig. 3f) (old Wt vs. old Tg, *p* = 0.0001).

In the relative pulsatility index, which reflected the diameter change, both the surface and the penetrating arteries showed the highest values in old Tg. The index value of the surface arteries was increased in old Tg compared to mid Tg and old Wt (Additional file 3) (mid Tg vs. old Tg, *p* = 0.0069; old Wt vs. old Tg, *p* = 0.0006). The penetrating arteries showed a significant decrease in the pulsatility index in old Wt compared to mid Wt and old Tg (mid Wt vs. old Wt, *p* < 0.0001, old Wt vs. old Tg, *p *< 0.0001). When combining the results from the relative pulsatility index and the pulsatility index, increased values in the surface arteries, penetrating arteries, and surface veins were noticeable in old Tg.

To examine whether the increased pulsatility indices observed in the old Tg group were related to CAA, the pulsatility index and vascular diameter were compared in arteries according to the presence or the absence of amyloid accumulation (Fig. 3g–l). In mid Tg, segments of surface arteries with CAA exhibit exaggerated movement and greater pulsatility index values than those without CAA (Fig. 3g, i) (mid CAA (−) vs. mid CAA (+), *p* = 0.0283). In contrast, an increased pulsatility index and an exaggerated movement were observed in both CAA and non-CAA segments in the old Tg group (Fig. 3h,i) (CAA, *p* = 0.0037; age, *p* = 0.0449; mid CAA(-) vs old CAA(+), *p* = 0.0015, two-way ANOVA with Bonferroni’s post hoc comparison). The reduction in diameter was particularly pronounced in segments with the amyloid deposits (Fig. 3k) (mid CAA(+) vs old CAA(+), *p* = 0.0478). However, in penetrating arteries, the effect of CAA on pulsatility index and diameters was not observed (Fig. 3j, l) (pulsatility index, interaction, *p* = 0.9471; CAA, *p* = 0.3849; age, *p* = 0.0838) (diameter, interaction, *p* = 0.8233; CAA, *p* = 0.0552; age, *p* = 0.1792). The heart rate, the origin of vascular pulsation, was not altered by the genotype (Additional file 3). Taken together, CAA progression leads to vSMC loss, reduced vascular diameter, and augmented vascular pulsation. Such pathological changes are more prominent in surface arteries with early Aβ accumulation than in penetrating arteries.

### Impairment of intramural periarterial drainage begins earlier and progressively worsens in APP/PS1 mice

**Fig. 4:**
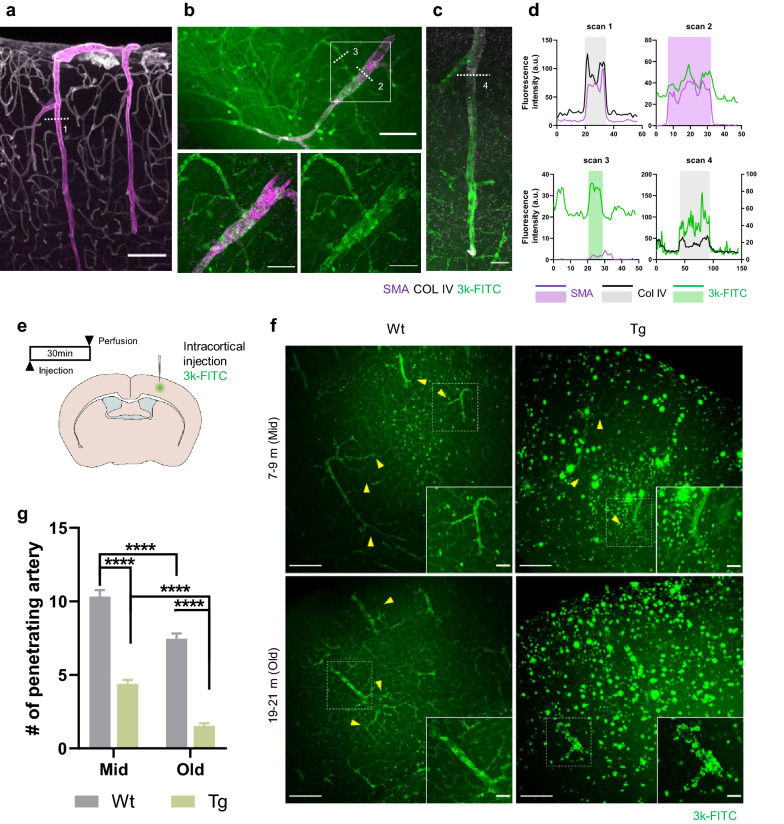
Early impairment of intramural periarterial drainage and progressive worsening in APP/PS1 mice. **a** Representative image of the vascular smooth muscle cells (vSMCs) and perivascular basement membrane (BM). vSMCs and BM were identified as smooth muscle actin (SMA) and collagen IV, respectively. Collagen IV shows a continuous cerebrovascular network of arteries, capillaries, and veins. Arteries are costained with SMA and collagen IV. Scale bar = 100 µm. **b** Representative image of 3 kDa FITC-dextran (3 k-FITC) distribution in the arteries (SMA) and capillaries in the parenchyma after somatosensory injection. The white-dashed box shows that the 3 k-FITC drains into arteries (SMA) and capillaries. **c** Representative image of 3 k-FITC and the BM (collagen IV). **d** Intensity profile analysis of the white dotted lines in images (**a–c**). vSMCs (SMA) are located inside the BM (collagen IV) layer (gray shaded area, top left). Note that 3 k-FITC is attached to the vSMCs, and the spatial distribution of 3 k-FITC is almost identical to the SMA signal (purple shaded area, top right). 3 k-FITC drains into the capillaries and exhibits a spatial distribution similar to that of the blood vessel (green shaded area, bottom left). The spatial distribution of 3 k-FITC is almost identical to that of collagen IV (gray shaded area, bottom right). This indicates the entry and exit of the 3 k-FITC through the BM. **e** Schematic illustration of intraparenchymal injection and perfusion schedule. **f** Representative image of the intramural periarterial drainage 3 k-FITC is located around the capillaries (yellow arrowhead) and arteries. Note that 3 k-FITC is rarely observed along the capillary in old Tg. The white-dashed boxes are magnified images of 3 k-FITC distribution around the arteries. Scale bar = 200 µm, 50 µm. **g** Quantification of the number of arteries with the 3 k-FITC signal. [*p* < 0.0001 (genotype), *p* < 0.0001 (age), two-way ANOVA) (mid Wt; n = 5, mid Tg; n = 5, old Wt; n = 6, old Tg; n = 5, 18 slices/mouse)]. All data are presented as the mean ± SEM. **p < 0.01, ***p < 0.001, ****p < 0.0001 two-way ANOVA with Bonferroni’s post hoc test

Pathological changes in periarterial BM, vSMCs, and vascular pulsation were observed and more pronounced by CAA progression, all of which are major factors contributing to IPAD impairment. We investigated how these dysfunctions of vasculature contribute to ISF drainage. 3 kDa FITC-dextran, which has a similar molecular weight to Aβ monomer, was injected into the somatosensory cortex, and the distribution pattern of dextran along the blood vessels was confirmed. To identify the spatial relationship between the tracer and vasculature, immunofluorescence was performed on the brain slices injected with tracers, and a line intensity profile was used (Fig. 4a–d).

BM identified by collagen IV forms a continuous layer covering the vSMCs of artery and capillaries (Fig. 4a, d). Tracers are distributed along the capillaries and artery in a similar pattern as collagen IV. At the capillary level, the fluorescence profile of the tracer had a boundary similar to that of the collagen IV, and at the arterial level, the tracer showed a similar profile to SMA (Fig. 4b, d). The collagen IV and tracer positions were almost identical indicating that the tracer entered and then drained along the BM (Fig. 4c, d).

IPAD was quantified by counting the number of penetrating arteries with tracer signals (Fig. 4e-g). SMA was used to identify whether the signal-positive vessel as an artery. In Tg mice, the number of tracer positive arteries decreased at the middle-aged compared with age-matched Wt (mid Wt vs mid Tg, *p* < 0.0001) and further decreased at old compared with mid Tg and age-matched Wt (mid Tg vs old Tg, *p* < 0.0001, old Wt vs old Tg, *p* < 0.0001). A decrease in IPAD was also associated with aging in Wt (Fig. 4g) (mid Wt vs old Wt, *p* < 0.0001; two-way ANOVA with Bonferroni’s post hoc comparison). However, the impairment of IPAD was more severe in Tg mice. In Wt mice, tracers appeared to be located in BM of the arteries and capillaries (Fig. 4f, yellow arrowhead), whereas in the old Tg mice, tracers rarely entered the BM of capillary despite the presence of signals in the parenchyma. When comparing the mid and old Tg, tracer distribution on the drainage path was partially preserved in mid Tg but was severely disrupted in old Tg. Tracers attached to the parenchymal plaques or arterial amyloid deposits rather than entering the BM. Although old Wt showed reduced clearance compared to mid Wt, the distribution of tracers in the BM was well preserved. Moreover, the cortical vascular density was not different across all experimental groups (Additional file 4).

### Decreased perivascular CSF influx occurs earlier and gradually progresses in APP/PS1 mice

**Fig. 5:**
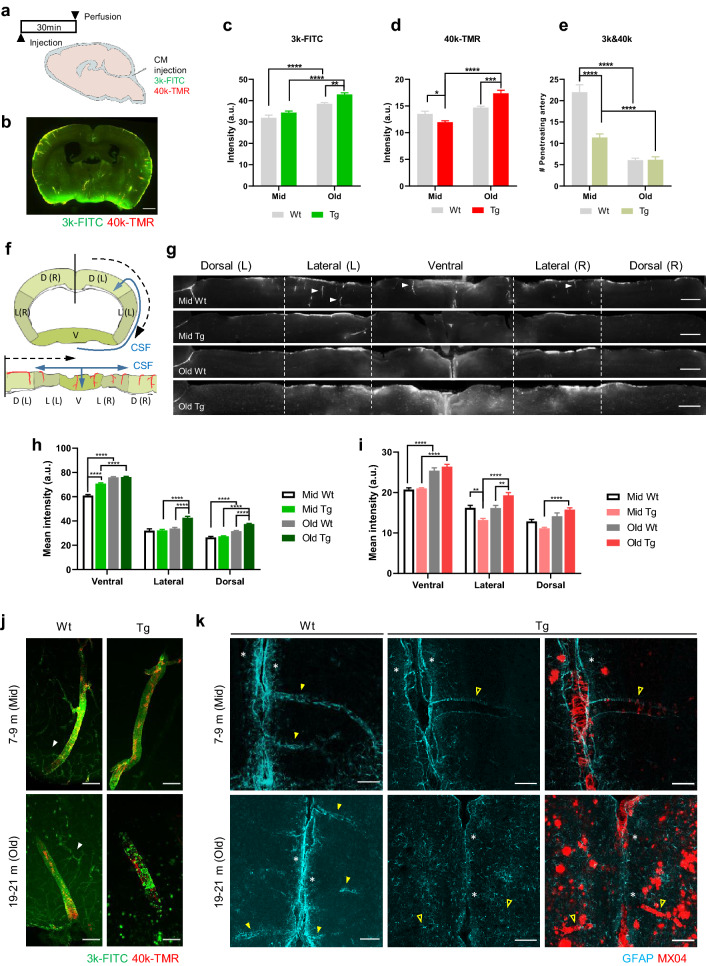
Early decrease in the perivascular CSF influx and gradual deterioration in APP/PS1 mice. **a** Schematic illustration of the cisterna magna (CM) injection and perfusion schedule. **b** Representative image of brain injected 3-kDa FITC-dextran (3 k-FITC) and 40-kDa TMR-dextran (40 k-TMR) via the cisterna magna. Scale bar = 100 µm. **c**, **d** Quantification of the CSF influx signal in 3 k-FITC (**c**) and 40 k-TMR (**d**). 3 k-FITC [*p* < 0.0001 (age), *p* = 0.0002 (genotype)]; 40 k-TMR [*p* < 0.0001 (age*genotype interaction), *p* < 0.0001 (age)]. **e** Quantification of the number of penetrating vessels with 3 k-FITC and 40 k-TMR signals. Note that the number of penetrating vessels with 3 k-FITC and 40 k-TMR decreases in the mid Tg and further decreases in old Tg. [*p* < 0.0001 (age), *p* < 0.0001 (genotype), *p* < 0.0001(age*genotype interaction)]. **f** Schematic illustration of the linearized cortical region of interest. Starting from the black mid-line, the cortex is linearized in the direction of the black dotted arrow (upper). The blue line represents the movement of CSF. D: dorsal, L: lateral, V: ventral, R: right, L: left. **g** Representative images of perivascular CSF influx within the brain cortex. Perivascular CSF influx was discovered along the penetrating vessels (white arrowheads). Note that the overall influx signal is increased in old Tg; however, the perivascular-specific signal is reduced. Scale bar = 100 µm. **h, i**. Regional differences in the CSF influx signal across the brain cortex in 3 k-FITC (**h**) and 40 k-TMR (**i**). 3 k-FITC {[ventral (*p* < 0.0001(age*genotype interaction), *p* < 0.0001 (age), *p* < 0.0001 (genotype)]; [lateral (*p* = 0.0001 (age*genotype interaction), *p* < 0.0001(age), *p* < 0.0001(genotype)]; [dorsal (*p* < 0.0001 (age*genotype interaction), *p* < 0.0001(age), *p* < 0.0001(genotype)]}. 40 k-TMR {[ventral (*p* < 0.0001(age); lateral (*p* < 0.0001 (age*genotype interaction), *p* < 0.0001(age)]; [dorsal (*p* = 0.0015 (age*genotype interaction), *p* < 0.0001(age)]}. **j** Representative images of tracer distribution in perivascular CSF influx. The 3 k-FITC is distributed along the arteries and capillaries in Wt (white arrowhead), but is not observed in the capillaries of Tg. Note that distribution of 3 k-FITC is disrupted in old Tg. Scale bar = 100 µm **k**. Representative images of the glia limitans around the pia mater and vessels. Astrocyte and amyloid plaque are identified using GFAP and MX04, respectively. Astrocytes (GFAP) are densely packed around the pia mater (white asterisk) and vessels (yellow arrowhead). Note that the astrocytes (GFAP) have almost disappeared around the vessels and are located around the parenchymal plaque in old Tg. Astrocytes are observed rarely around blood vessels with excessive amyloid deposition (yellow line arrowhead). Scale bar = 100 µm. (mid Wt = 9, mid Tg = 9, old Wt = 8, old Tg = 8, 7 slices/mouse). All data are presented as the mean ± SEM. ** *p* < 0.01, *** *p* < 0.001, **** *p* < 0.0001, two-way ANOVA with Bonferroni’s post hoc test

Perivascular CSF influx exhibits pulsatile flow in accordance with vascular pulsation and enters the brain along the pial-glia BM [17, 21, 33]. Two types of dextran with different molecular weights were delivered through the CM to investigate the perivascular CSF influx (Fig. 5a, b). The fluorescence signal was quantified in two ways by dividing the entire brain and perivascular compartments. The fluorescence signals from the brain slices were generally the highest in old Tg (3 k-FITC, age, *p* < 0.0001; genotype, *p* = 0.0002; 40 k-TMR, interaction, *p *< 0.0001; age, *p *< 0.0001). The signal was higher in old Tg than mid Tg [mid Tg vs. old Tg, *p* < 0.0001 (3 k-FITC), *p* < 0.0001 (40 k-TMR)] and also higher than age-matched Wt mice (Fig. 5c,d) [old Wt vs. old Tg, *p* = 0.0051 (3 k-FITC), *p *= 0.0001 (40 k-TMR)]. In middle-aged mice, the fluorescent signal was lower in Tg than in age-matched Wt mice (Fig. 5d) (mid Wt vs. mid Tg, *p* = 0.0442 (40 k-TMR)). Regarding the 3-kDa tracer, the parenchymal signal was higher in the old Wt than in the mid Wt group, but not for the 40-kDa tracer (Fig. 5c, d) (mid Wt vs old Wt, *p* < 0.0001 (3 k-FITC)). In summary, the influx signal was decreased in mid Tg, and then increased in old Tg. To specify the perivascular CSF influx from the whole brain signal, we counted the number of penetrating vessels with tracer signals (Fig. 5e) (interaction, *p* < 0.0001; age, *p* < 0.0001; genotype, *p* < 0.0001). The number of penetrating vessels was lower in the mid Tg than in the age-matched Wt group and was further reduced in the old Tg group (mid Wt vs. mid Tg, *p* < 0.0001; mid Tg vs. old Tg, *p *= 0.0024). This decrement was also associated with aging, comparing old Wt with mid Wt (Fig. 5e) (mid Wt vs. old Wt, *p* < 0.0001).

Since there was a discrepancy in the pattern of signal changes between the perivascular compartments and the parenchyma, we tried to determine which brain regions contributed to the changes in parenchymal influx and investigated what changes occurred around the penetrating vessel. The tracers injected into the CM initially gathered around the circle of Willis and flowed mainly along the middle cerebral arteries [29, 31]. Thus, CSF influx occurred in the order of the ventral, lateral, and dorsal parts of the cortex, and this pattern was also similar in the tracer movement. Based on these previous findings, the ROI was divided into dorsal, lateral, and ventral parts and linearized to identify how the tracer was distributed in each region (Fig. 5f). In the mid Wt group, perivascular CSF influx around the penetrating vessel was well observed in overall regions (Fig. 5g, white arrowhead), but in the old Tg mice, there was little perivascular CSF signal, though the parenchymal tracer signal was high (Fig. 5g).

To determine regional differences of parenchymal influx, the dextran signals in the ventral, dorsal, and lateral regions were compared (Fig. 5h,i). In old Tg mice the brightest signal was detected across the brain regardless of tracer type. When comparing Tg mice with age-matched Wt mice, mid Tg showed reduced signal in the dorsal and lateral areas [mid Wt vs. mid Tg, *p* = 0.0015 (40 k-TMR, lateral)], while old Tg showed an increased signal in the dorsal and lateral areas [old Wt vs. old Tg, *p *= 0.0017 (40 k-TMR, lateral), *p* < 0.0001 (3 k-FITC, lateral), *p *< 0.0001 (3 k-FITC, dorsal)]. Within the Tg groups, the signal in the old Tg was higher in the whole brain than mid Tg (mid Tg vs. old Tg, *p* < 0.0001 (3 k-FITC; ventral, lateral, dorsal), *p* < 0.0001 (40 k-TMR, ventral, lateral, dorsal). In Wt groups, the increased signals were mainly observed in the ventral area of old Wt mice (mid Wt vs old Wt, *p* < 0.0001 (3 k-FITC, ventral, dorsal), *p* < 0.0001 (40 k-TMR, ventral). When comparing mid and old age within the same genotype, increased ventral signal was generally observed in both old Wt and old Tg mice (Fig. 5h, i).

We further examined the distribution of tracers around penetrating arteries and surrounding capillaries to investigate whether pathological changes of arteries influence the CSF influx pattern. The tracer showed a stripe pattern similar to morphology of the BM covering the vSMCs and was continuously distributed along the arteries and the capillaries in Wt mice (Fig. 5j, white arrowhead). In the mid Tg mice showing reduced perivascular CSF influx, a trace distribution pattern around the penetrating artery was well observed compared with age matched Wt mice, but this signal did not continue to the capillaries. In old Tg mice with severely impaired perivascular CSF influx, tracers were discontinuously distributed and accumulated around amyloid deposition at the arterial level and were not detected in capillaries (Fig. 5j). Despite the decrease in perivascular CSF influx observed in old Wt, the distribution of tracers around vessels was well maintained similar to mid Wt.

Although the tracer’s entrance into the penetrating arteries and capillaries was interrupted, the increased tracer signal in old Tg mice raised the possibility of other pathways being present. We speculated that the glia limitans was compromised allowing the tracer to access the parenchyma without moving along the BM. To confirm this hypothesis, we examined the distribution of astrocytes around the blood vessels and the pia mater. Similar to the previous reports, astrocytes were densely located around pia mater and the blood vessels, which was consistently observed in mid Wt, mid Tg, and old Wt mice (Fig. 5k, white asterisk, and yellow arrowhead). In the old Tg mice, the astrocytes decreased around the pia mater and blood vessels, and instead increased around the plaque, indicating that the glia limitans was damaged with AD progression [47, 50]. In addition, astrocytes increased around the parenchymal plaque, but were rarely observed around the vessels with amyloid deposits (Fig. 5k, yellow line arrowhead). These findings imply that the perivascular CSF influx decreases from middle age and further decreases in old age in Tg mice, and non-perivascular CSF influx occurs in old Tg mice with disruption of the glia limitans.

### The amyloid burden is lower in areas with higher CSF influx

**Fig. 6:**
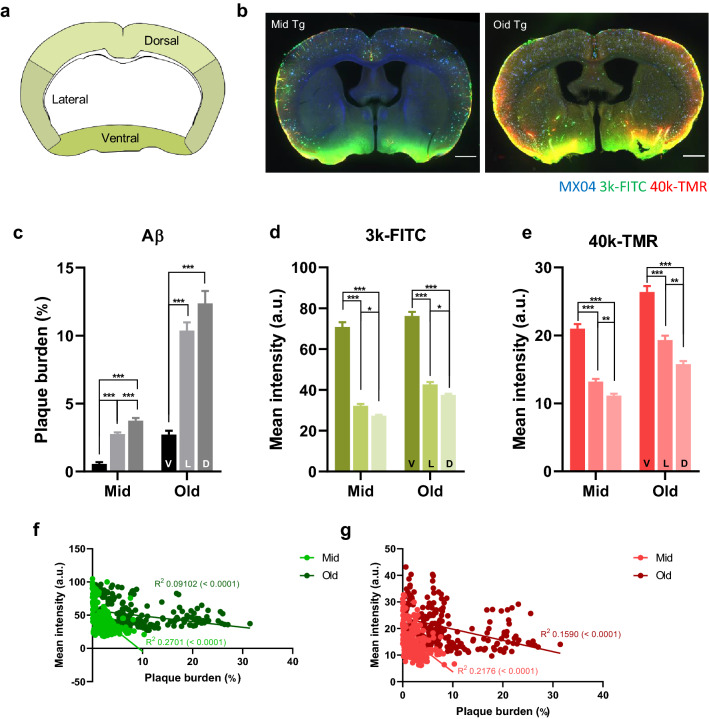
Lower amyloid burden in areas with higher CSF influx. **a** Brain template for analysis of the regional differences. The dorsal, lateral, and ventral regions are marked with different colors. **b** Representative brain images with amyloid plaque (blue, MX04), 3 k-FITC (green), and 40 k-TMR (red) signals in mid and old Tg mice. Scale bar = 100 µm. **c** Regional differences in plaque burden. Note that the amyloid burden increases from the ventral (V) toward the lateral (L) and dorsal cortices (D) in both mid and old Tg (mid, *p* < 0.001; old, *p* < 0.001, one-way ANOVA). **d, e** Regional differences in the parenchymal signals of 3 k-FITC (**d**) and 40 k-TMR (**e**). Note that the tracer signal is the highest in the ventral region and decreases toward the lateral (L) and dorsal (D) cortices. [3 k-FITC; mid, *p* < 0.001; old, *p* < 0.001] [40 k-TMR; mid, *p* < 0.001; old, *p* < 0.001]. **f, g**. Linear regression between amyloid burden and influx signals of 3 k-FITC (**f**) and 40 k-TMR (**g**) in mid and old Tg mice. Note the negative linear correlation between the amyloid plaque burden and influx, which is consistent irrespective of the tracer weight and Alzheimer’s disease progression. [3 k-FITC (mid, *p* < 0.0001, R^2^ = 0.2701; old, *p* < 0.0001, R^2^ = 0.09102)]; [40 k-TMR (mid, *p* < 0.0001, R^2^ = 0.2176; old, *p* < 0.0001, R^2^ = 0.1590)] (n = 9, mid Tg, n = 8, old-Tg, 7 slices/mouse). All data are presented as the mean ± SEM. *p < 0.05, **p < 0.01, ***p < 0.001, ****p < 0.0001 One-way ANOVA with Tukey’s post hoc test

We also found that the influx of tracers remained relatively high in the ventral part of the brain regardless of aging or AD progression (Fig. 6a, b). We assumed that amyloid accumulation occurs less in the ventral area where the CSF influx is relatively active. To test whether the CSF influx pattern is correlated with amyloid plaque burden, the relationship between amyloid burden and tracer signal was analyzed in dorsal, lateral, and ventral parts of the brain in mid and old Tg mice (Fig. 6c–g). Tracer signals increased more toward the dorsal, lateral, and ventral parts [3 k-FITC; ventral vs lateral, *p* < 0.001 (mid, old); ventral vs dorsal, *p* < 0.001 (mid, old); lateral vs dorsal, *p* = 0.02 (mid, old), one-way ANOVA with Tukey’s post hoc comparison] [40 k-TMR; ventral vs lateral, *p* < 0.001 (mid, old); ventral vs dorsal, *p* < 0.001 (mid, old); lateral vs dorsal, *p* = 0.007 (mid), *p* = 0.001 (old)], whereas the amyloid burdens decreased, and this pattern was same in both mid and old Tg (Fig. 6c–e, Additional file 5) [amyloid; ventral vs lateral, *p* < 0.001 (mid, old); ventral vs dorsal, *p* < 0.001 (mid, old); lateral vs dorsal, *p* < 0.001(mid)]. There was a strong negative correlation between amyloid burden and tracer signal, which was consistent regardless of the molecular weights of the tracers and AD stages (Fig. 6f, g) [3 k-FITC; R^2^ = 0.2701, *p* < 0.0001 (mid); R^2^ = 0.0912, *p* < 0.0001 (old)] [40 k-TMR; R^2^ = 0.2176, *p* < 0.0001(mid); R^2^ = 0.1590, *p* < 0.0001(old)]. These results indicate that amyloid accumulation is mitigated in areas with a higher CSF influx.

## Discussion

**Fig. 7:**
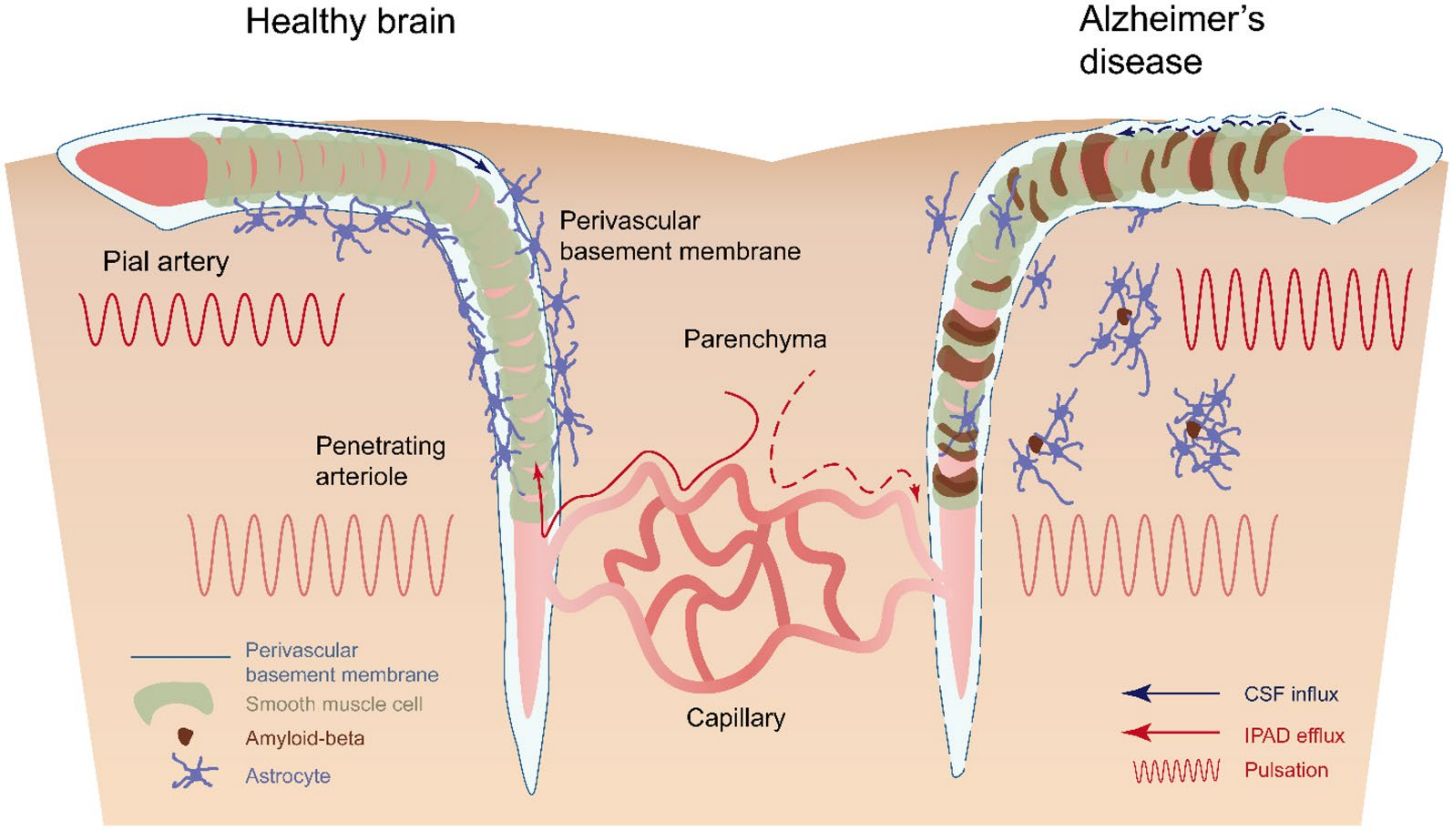
Pathological changes in the vasculature and perivascular clearance in Alzheimer’s disease with CAA: Schematic summary. Cerebrospinal fluid (CSF) influx (blue arrow) and intramural periarterial drainage (IPAD) efflux (red arrow) occurring along the perivascular basement membrane (BM) under the influence of vascular pulsation in the healthy young brain (left). Astrocytes surround the perivascular BM to form glia limitans. Amyloid accumulation in the vascular wall leads to structural changes in the BM, loss of vascular smooth muscle cells (vSMCs), and augmented vascular pulsation in the Alzheimer’s disease brain (right). This vascular dysfunction inhibits perivascular CSF influx and IPAD efflux, and the pathological changes in the vasculature and clearance further worsen with the progression of CAA. The astrocytes located around the blood vessels and pia mater move around the parenchymal Aβ plaque, thus the glia limitans is also compromised

In this study, we investigated the effect of CAA progression on the structure and function of vasculature and perivascular clearance. Previously, CAA was assumed to be a result of a failure of perivascular clearance; however, the exact effect of CAA on the clearance has not been well documented. In the present study, we found that amyloid deposition on the vascular wall destroyed the periarterial BM, vSMCs, and altered vascular pulsation, and then subsequently impaired the perivascular clearance, IPAD, and perivascular CSF influx (Fig. 7). Furthermore, as CAA progressed, pathological changes related to arteries became more pronounced and both types of perivascular clearance worsened.

We highlight that there were differences in pathological changes depending on the cerebrovascular tree. Even though the Aβ is produced in the brain parenchyma, we found that the amyloid accumulation occurs earlier and more in the surface arteries (Additional file 2). In addition to CAA susceptibility, changes in the BM, vascular diameter, and pulsation were more pronounced in the surface arteries than the penetrating arteries. More advanced pathological changes observed in the surface arteries imply that CAA contributes to these changes. These results are consistent with the previous reports demonstrating that CAA is more frequently discovered in leptomeningeal arteries in patients with Alzheimer’s disease [7, 13]. Interestingly, at the point where the surface arteries become penetrating arteries, much more Aβ was accumulated in surface arteries than in adjacent penetrating arteries (Fig. 2d). One possible explanation is the active myogenic tone of penetrating arteries (Fig. 3b) prevents the accumulation of Aβ. Unlike surface arteries, which are located in subarachnoid space and have low resistance, the penetrating arteries are surrounded by the brain parenchyma and thus have higher resistance against external tissue and are known to generate much stronger myogenic tone [25, 53]. Also, more amyloid accumulated in the deep cortex than in superficial positions within the same penetrating artery. This accumulation pattern is partially explained by a recent study that showed the portion of the penetrating artery close to the pia mater exhibits the strongest vascular movement during voluntary movement such as walking [14]. Taken together, the strong myogenic tone of the penetrating arteries impedes amyloid accumulation, while the surface arteries accumulate amyloid deposition faster, leading to changes in the major components of perivascular clearance and clearance impairment.

Our study demonstrated that amyloid deposition on surface arteries disrupts the integrity of the BM and altered the morphology. The integrity of the BM was compromised by the perforation of the BM layer where amyloid was accumulated (Fig. 1b). Disruption of BM integrity allowing Aβ protein to penetrate and accumulate outside of the BM was detected from middle-aged onward. Nevertheless, amyloid mostly accumulated inside of the BM even in old Tg mice, and furthermore, the morphology of the BM was changed according to the shape of CAA (Fig. 1b). Continued morphological modification due to additional amyloid accumulation means that Aβ is mainly drained inside of the BM through IPAD, and this finding supports the IPAD hypothesis [3, 35]. In contrast, in penetrating arteries, the BM integrity disruption was more pronounced rather than morphological changes.

BM is the major component of the perivascular compartments and is known to be a pathway for solute efflux and CSF influx in IPAD and the perivascular CSF influx respectively, so alterations in the BM can also interfere with influx and efflux [17, 37]. The detailed structure of the perivascular compartments was recently revealed and it is composed of at least three layers; BM of pia-glia, BM of vSMCs, and that of endothelial cells [17, 35]. CSF influx occurs at the potential space between vSMCs BM and pial-glial BM, and solute efflux occurs inside the BM of vSMCs, illustrating two types of perivascular clearance occur at different layers with opposite directions [9]. However, these two layers are extremely close together such that the change of the shape and the integrity of one layer would influence the other. In particular, the morphological change and integrity disruption of the BM covering vSMCs shown in our study affect two layers concurrently, thereby induce decreasing the efflux of solutes and also the influx of CSF into the brain.

We found that both types of perivascular clearance were impaired in middle-aged Tg mice and further impaired in old age. At middle-age, CAA of the surface arteries became clear and other pathological changes, such as augmented hemodynamic response, also became apparent [23, 24]. The tracer injected into parenchyma was distributed in the BM of the capillaries and penetrating arteries (Fig. 4b). In mid Tg, despite the impairment of perivascular clearance, the tracer movement along the BM was well preserved, while in old Tg, the clearance was further impaired and the distribution of tracers located in clearance route was significantly disrupted. Similarly, the perivascular CSF influx pattern was preserved in mid Tg but severely impaired in old Tg. Tracers delivered through the cisterna magna were continuously distributed around the blood vessels in Wt and mid Tg mice, but in old Tg mice, the distribution pattern of tracers was discontinuous and adhered around CAA. These findings indicate that CAA-induced changes in the BM have a greater impact on the influx and efflux of CSF. On the other hand, the influx or efflux patterns themselves did not significantly change in old Wt mice despite the decrease in perivascular clearance. This may be related to changes in the composition of the BM during aging [18]. Hawkes et al. reported that the components of the BM, including laminin, fibronectin, and perlecan, were significantly changed by aging and more so in capillaries than in large-diameter vessels.

CAA also affected vascular morphology and movement. The surface artery maintains a constant vascular diameter compared to the surface vein. We observed that a reduction in vascular diameters was associated with CAA progression in Tg mice groups, mainly in surface arteries and more prominently in CAA-positive segments (Fig. 3k). The vascular diameter is the most critical factor in vascular resistance, and the surface arteries are responsible for 21% of the total vascular resistance [19]. Reducing the diameter of the surface arteries would markedly increase vascular resistance, presumably further exacerbating brain damage in AD [44]. However, this decrease was not detected in penetrating arteries where amyloid was deposited relatively slowly.

To the best of our knowledge, this study is the first to demonstrate that CAA was associated with increased vascular pulsation, and this increase was initially observed in surface arteries of mid Tg mice (Fig. 3i). In middle age, the increase in pulsatility index was evident only in the segments with CAA, but in old age, this increase extended throughout the surface arteries. We propose that the augmented pulsation in the CAA-positive segment was related to vSMC loss, as vSMCs and amyloid accumulation showed opposite distributions (Fig. 2c, d). The increased pulsation would be the result of the loss of the basal vascular tone caused by damage to the vSMCs. The vascular pulsation is primarily driven by the heartbeat, and vSMCs buffer the pressure shock of the pulse during the systolic phase by maintaining the constant vascular tone during the diastolic phase [8, 33, 46]. Blood vessels without vSMCs become floppy, passively reflecting the heartbeat, and thus exaggerated vascular movement results in increasing the pulsatility index. Moreover, this enhanced pulsation due to loss of vSMCs may be a preliminary stage of microbleeds which is commonly accompanied by CAA [12, 15, 50]. When vSMC loss occurs and vascular pulsation increases, the luminal side becomes vulnerable to pressure and is prone to rupture. This has important implications for understanding the role of vSMCs in vascular pulsation and the relationship between CAA and microbleeds.

Previous studies have demonstrated that perivascular CSF influx and vascular pulsation are closely related to the regulation of pulsation by vasopressor drugs and surgical procedures [21, 27, 33]. However, in this study, even with increased vascular pulsation, the perivascular CSF influx was decreased in old Tg mice. This discrepancy may be caused by damaged vascular components that hamper the pulsation effect on the perivascular CSF influx. The exaggerated vascular movement would also lead to the loss of synchronous or harmonized movement of vessel walls and CSF influx. Similarly, a study showed that vascular movement increased in hypertension, but rather, CSF influx decreased as the portion of backflow increased around the artery [33].

In addition, further research is needed to investigate the role of the vascular components in vascular pulsation. In this study, we found that there is a change in pulsatility index with aging and AD progression without heart rate change, which is the cause of pulsation. As in the previous report, a decrease in the pulsatility index of the penetrating arteries and perivascular CSF influx was observed in the old Wt mice [27]. However, this decrease in the pulsatility index of the penetrating arteries cannot be explained by the heart rate or vSMCs, which means that other vascular components may be involved in this change. Previous studies have been conducted mainly in young healthy mice with intact vascular components, therefore the role of vascular components is relatively underestimated. To understand the perivascular clearance in other pathological conditions related to vascular dysfunction, it is necessary to reveal what the vascular components contribute.

Vascular pulsation promotes the opportunity for solutes to enter the IPAD pathway, as increased heart rate was found to facilitate the volume distribution of the tracer in parenchyma [16, 40]. Recent studies have shown that not only vascular pulsation but also another type of vascular movement, called vasomotion, is also involved in IPAD. Vasomotion is caused by the synchronized action of vSMCs and is observed at a lower frequency independent of pulsation and respiration [4, 36, 45]. In our study, we did not directly examine the vasomotion, but a recent finding showed that IPAD and vasomotion are impaired in middle-aged Tg mice with vSMC loss [45]. This study is consistent with our findings in that the IPAD impairment occurs in mid-age or earlier, and vSMCs are important for perivascular clearance. Further research is needed to investigate how particular types of vascular movements, such as vasomotion and pulsation, participate in both types of perivascular clearance and their working mechanisms.

Interestingly, a decrease in perivascular CSF influx and IPAD was observed both in aging and AD, but the changes in the major components of perivascular clearance were different in each situation. The pulsatility index of the penetrating artery, for example, which is important for influx or efflux into the parenchyma, decreased in old Wt not changed in old Tg when compared to genotype matched middle-aged mice respectively. In Wt group, clearance was suppressed with the decreased pulsatility index of penetrating artery in old age, but the distribution of tracers in the BM did not change significantly unlike Tg mice. In other words, perivascular clearance occurs in different ways in aging and AD, and more serious and earlier changes appear in AD with amyloid accumulation.

We found that amyloid burden and CSF influx signals show a negative relationship independent of the tracer size and the age of the mouse. Especially, the amyloid burden was the lowest in the ventral region where CSF entrance was the highest. These findings support the possibility that CSF movement is involved in Aβ clearance. Unlike cortical vessels located in the dorsal and lateral regions, the penetrating vessels in the ventral region are larger and have two meningeal layers, and this difference may also be associated with low amyloid burden [46]. In addition, the recent finding that lymphatic drainage in the dura mater predominantly occurs in the ventral part rather than the dorsal part also appears to be related to the low amyloid burden in the ventral region [2]. Whether meningeal lymphatic vessels involve in Aβ clearance should be further investigated.

Moreover, we found that the glia limitans was involved in parenchymal influx at the late stages of AD. In contrast to the perivascular tracer signal, the fluorescence signal from brain slices decreased in the mid Tg and increased in old Tg. The latter increased signal occurs through the non-perivascular pathway and is accompanied by disruption of glia limitans, the outermost boundary upon the CSF influx. Astrocytes are well distributed around the blood vessels and pia mater of Wt and mid Tg, but in old Tg, they are mainly observed around plaque instead of blood vessels; thus, disruption of glia limitans eventually influences CSF inflow. Glia limitans also work as a barrier for the brain, so this disruption can cause immune problems, and hence the pial barrier changes that occur in AD need further investigation.

One of the limitations of our study was that we did not see the contribution of AQP4 in APP/PS1 mice [27, 32]. Instead, we focused on the common factors of IPAD and the perivascular CSF influx involved upstream of the CSF influx before reaching AQP4. A recent human and rodent post-mortem study reported the loss of perivascular AQP4 in patients with AD and transgenic mice model [49, 51, 52]. AQP4 is known to be located in perivascular astrocyte endfeet; however, AQP4 is highly expressed around amyloid plaques in the brain parenchyma. Similarly, we have also shown that the position of the astrocytes changed from the vascular and pia mater to the plaque, suggesting that AQP4 localization was also affected by the loss of astrocytes. Thus, the major parameters related to perivascular clearance presumably interfered within AD. In this regard, there are some debates about IPAD and glymphatic system studies. For example, the role of AQP4 on parenchymal clearance and the physiological relevance of the glymphatic system is still under debate [1]. Conflicting results exist whether AQP4 function affects parenchymal clearance. Although AQP4 is known to involve the perivascular CSF influx and convective transport in the parenchyma [1, 20], a recent study has shown that the transport of tracer in the brain is mainly by diffusion rather than convection, and CSF influx is not significantly impaired in AQP4 gene deleted mice [42].

Another pitfall of our study is that it is difficult to isolate only the IPAD effect from the time window used in this experimental condition, as IPAD occurs very quickly within 5 to 10 min followed by CSF-ISF interactions. Our study focused on the changes in the common key components in the pathological condition of AD rather than differences between two systems, therefore further studies are further warranted to elucidate these differences.

## Conclusion

Our research investigated how CAA contributes to Aβ clearance with regard to vascular components and vascular movement. As CAA progresses, pathological changes in the blood vessels become more apparent, and perivascular clearance further worsens. In contrast to amyloid plaque accumulated in the parenchyma that causes synaptic and neuronal loss and inflammation, the effect of Aβ on the vascular wall is underestimated. However, vascular damage accompanied by CAA growth appears to cause a vicious cycle by aggravating the Aβ clearance system. Prevention and reversal of vascular damage, which is considered less important, needs to be actively reviewed in AD patients.

## Supplementary information

**Additional file 1: Figure S1.** Mouse information used in each experiment.

**Additional file 2: Figure S2.** Amyloid accumulation occurs earlier and more in the surface arteries.

**Additional file 3: Figure S3.** Augmented pulsation is also observed in the relative pulsatility index.

**Additional file 4: Figure S4.** Vascular density was not changed with ageing and AD progression.

**Additional file 5: Figure S5.** Regional differences in CSF influx and amyloid burden.

## Data Availability

The data that support the findings of this study are available from the corresponding author upon reasonable request.

## Abbreviations

Aβ: amyloid-beta
AD: Alzheimer’s disease
AQP4: aquaporin 4
BM: basement membrane
CAA: cerebral amyloid angiopathy
CM: cisterna magna
CSF: cerebrospinal fluid
IPAD: intramural periarterial drainage
ISF: interstitial fluid
MX04: methoxy-04
PBS: phosphate-buffered saline
ROI: region of interest
SMA: smooth muscle actin
Tg: transgenic
vSMC: vascular smooth muscle cell
Wt: wild-type
