# Supplementary material for: Cerebral amyloid angiopathy aggravates perivascular clearance impairment in an Alzheimer’s disease mouse model
Source: Acta Neuropathol Commun. 2020 Nov 5;8:181. doi: 10.1186/s40478-020-01042-0 (PMC7643327; doi:10.1186/s40478-020-01042-0)
Supplement: Supplementary file 1 — Additional file 1: Figure S1. Mouse information used in each experiment. [file 40478_2020_1042_MOESM1_ESM.pdf]

Experiment	Used mouse per group	Male and female balance
**Immunofluorescence**<br>**Male 8, Female 8 (n=16)**<br>**(50% : 50%)**	Mid Wt = 4<br>Mid Tg = 4<br>Old Wt = 4<br>Old Tg = 4	Male 2 female 2 (50% : 50%)<br>Male 2 female 2 (50% : 50%)<br>Male 2 female 2 (50% : 50%)<br>Male 2 female 2 (50% : 50%)
**Vascular pulsation**<br>**Male 11, Female 14 (n = 25)**<br>**(44% : 56%)**	Mid Wt = 6<br>Mid Tg =6<br>Old Wt = 6<br>Old Tg = 7	Male 3 female 3 (50% : 50%)<br>Male 2 female 4 (33% : 67%)<br>Male 3 female 3 (50% : 50%)<br>Male 3 female 4 (43% : 57%)
**Intraparenchymal injection**<br>**Male 11, Female 10 (n = 21)**<br>**(52% :48%)**	Mid Wt =5<br>Mid Tg =5<br>Old Wt =6<br>Old Tg = 5	Male 3 female 2 (60% : 40%)<br>Male 3 female 2 (60% : 40%)<br>Male 2 female 4 (33% : 67%)<br>Male 3 female 2 (60% :40%)
**Cisterna magna injection**<br>**Male 18, Female 16 (n = 34)**<br>**(53% : 47%)**	Mid Wt =9<br>Mid Tg =9<br>Old Wt =8<br>Old Tg =8	Male 5 female 4 (56% : 44%)<br>Male 5 female 4 (63% : 37%)<br>Male 4 female 4 (50% : 50%)<br>Male 4 female 4 (50% : 50%)
**Total n = 96**		Male 48, female 48 (50% :50%)

Additional file 1. Mouse information used in each experiment
