# Supplementary material for: Cerebral amyloid angiopathy aggravates perivascular clearance impairment in an Alzheimer’s disease mouse model
Source: Acta Neuropathol Commun. 2020 Nov 5;8:181. doi: 10.1186/s40478-020-01042-0 (PMC7643327; doi:10.1186/s40478-020-01042-0)
Supplement: Supplementary file 2 — Additional file 2: Figure S2. Amyloid accumulation occurs earlier and more in the surface arteries. [file 40478_2020_1042_MOESM2_ESM.pdf]

Additional file 2

a 7-9 m (Mid) Tg

b 19-21 m (Old) Tg

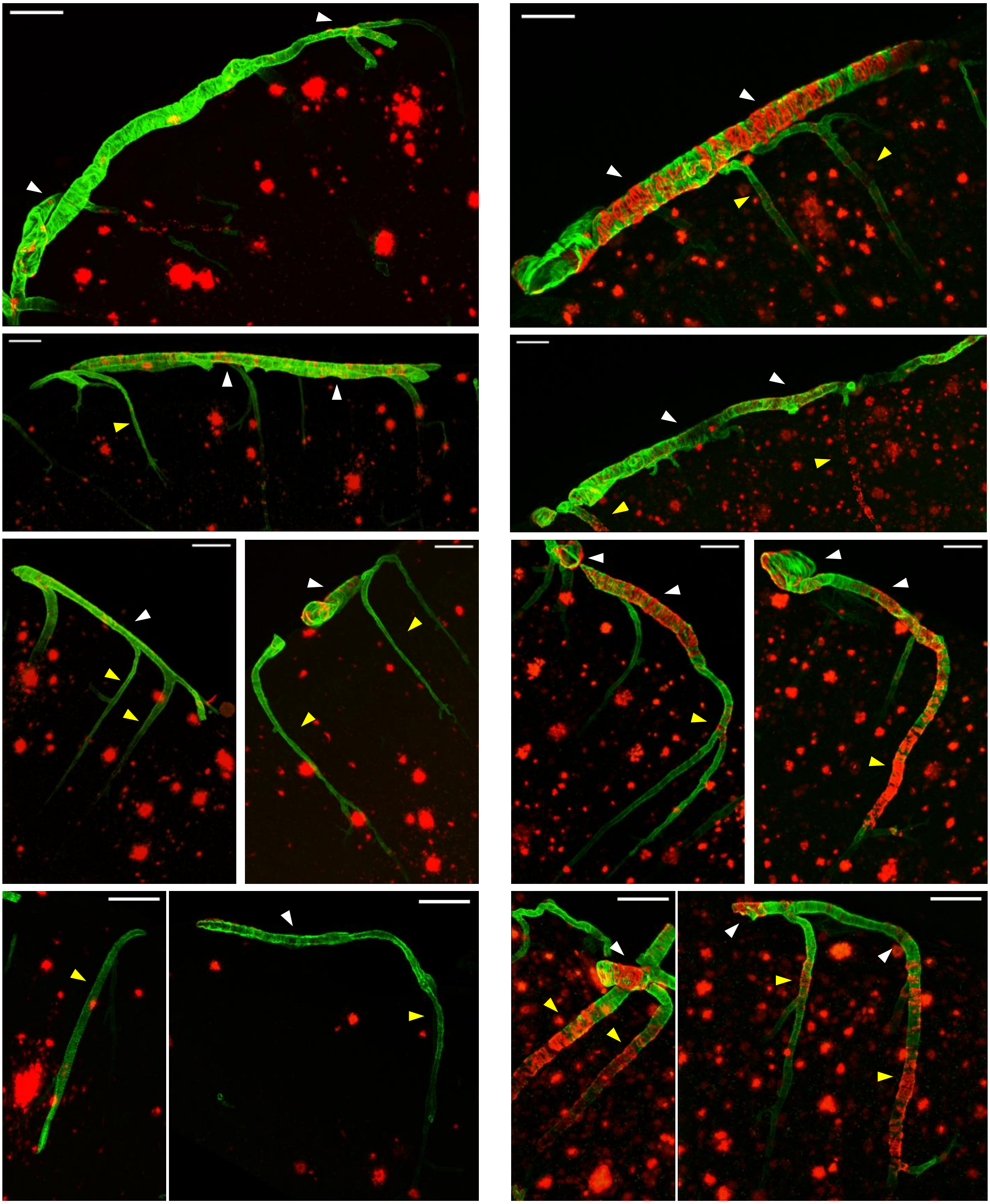

MX04 SMA

Additional file 2. Amyloid accumulation occurs earlier and more in the surface arteries

a-b. Representative images of CAA across the arterial vascular tree in mid and old Tg mice. SMA was used to identify arteries. Amyloid plaques (MX04) mainly accumulated in the surface artery (white arrowhead) of mid Tg (**a**). In old Tg, CAA was clearly observed in both the surface (white arrowhead) and penetrating artery (yellow arrowhead) (**b**). Among the penetrating arteries, large diameter vessels tend to accumulate more Aβ plaque. Within the same penetrating arteries, Aβ tends to accumulate more in a segment located deeper than the surface (**b**). Scale bar = 100µm.
