# Supplementary material for: Cerebral amyloid angiopathy aggravates perivascular clearance impairment in an Alzheimer’s disease mouse model
Source: Acta Neuropathol Commun. 2020 Nov 5;8:181. doi: 10.1186/s40478-020-01042-0 (PMC7643327; doi:10.1186/s40478-020-01042-0)
Supplement: Supplementary file 3 — Additional file 3: Figure S3. Augmented pulsation is also observed in the relative pulsatility index. [file 40478_2020_1042_MOESM3_ESM.pdf]

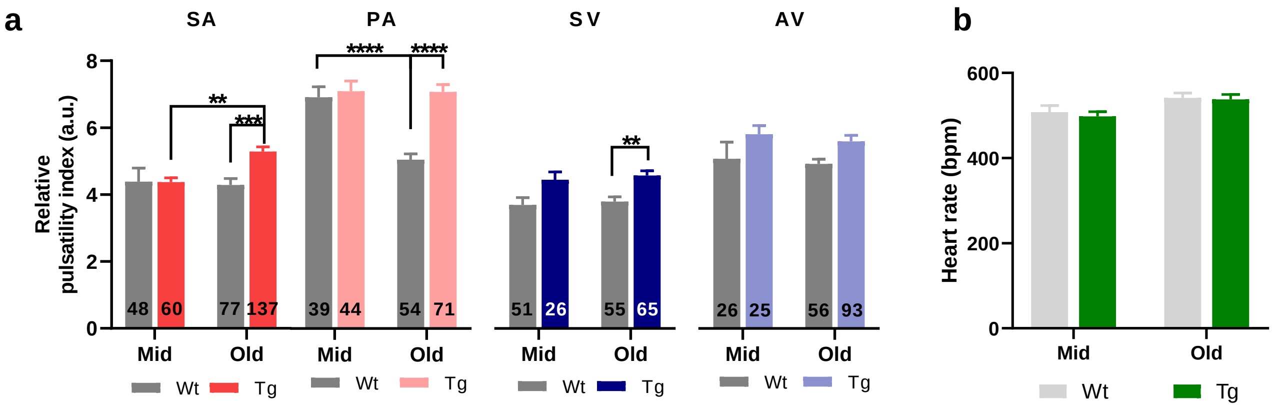

Additional file 3. Augmented pulsation is also observed in the relative pulsatility index

a. Relative vascular pulsation along with the cerebrovascular tree. Note that the increased relative pulsatility index is remarkable in surface and penetrating arteries of old Tg mice. [Surface arteries (SA), $p = 0.0192$ (genotype*age interaction), $p = 0.0232$ (genotype); Penetrating arteries (PA), $p = 0.0003$ (genotype*age interaction), $p = 0.0002$ (age), $p < 0.0001$ (genotype); Surface vein (SV), $p < 0.0001$ (genotype), two-way ANOVA], (mid Wt =6, mid Tg =6, old Wt =6, old Tg =7). **b.** Comparison of heart rate. ($p < 0.0064$ (age), two-way ANOVA), (n = 8, each group). All data are presented as the mean ± SEM. ** $p < 0.01$, *** $p < 0.001$, **** $p < 0.0001$. Two-way ANOVA with Bonferroni's post hoc test.
