# Supplementary material for: Cerebral amyloid angiopathy aggravates perivascular clearance impairment in an Alzheimer’s disease mouse model
Source: Acta Neuropathol Commun. 2020 Nov 5;8:181. doi: 10.1186/s40478-020-01042-0 (PMC7643327; doi:10.1186/s40478-020-01042-0)
Supplement: Supplementary file 4 — Additional file 4: Figure S4. Vascular density was not changed with ageing and AD progression. [file 40478_2020_1042_MOESM4_ESM.pdf]

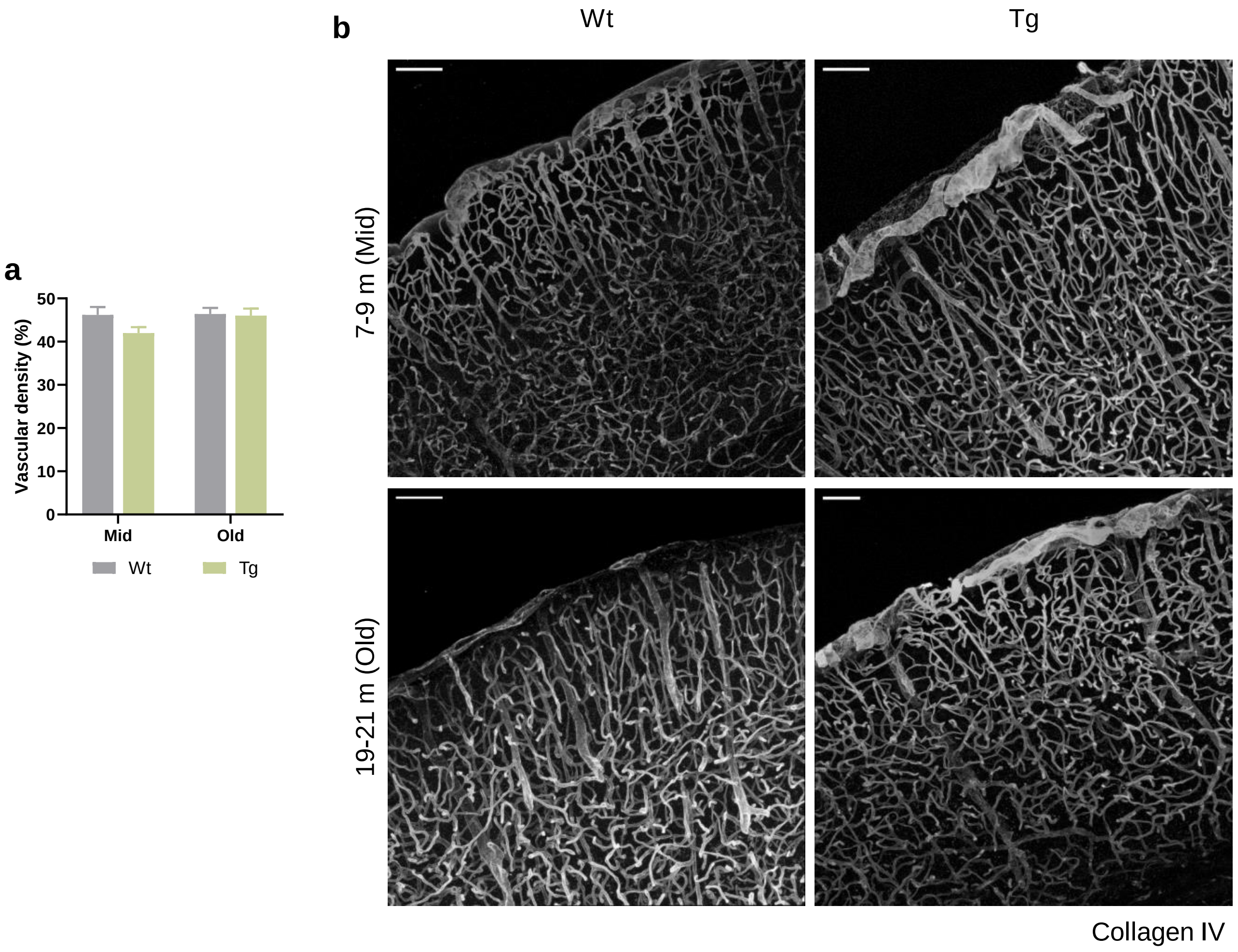

Additional file 4. Vascular density was not changed with ageing and AD progression

a. Comparison of vascular density. No significant difference was observed (interaction, $p = 0.2455$; age, $p = 0.2014$; genotype, $p = 0.1666$) (all groups n = 4). **b.** Representative images of cortical vascular density. Scale bar = 100μm. All data are presented as the mean ± SEM.
