# Supplementary material for: Cerebral amyloid angiopathy aggravates perivascular clearance impairment in an Alzheimer’s disease mouse model
Source: Acta Neuropathol Commun. 2020 Nov 5;8:181. doi: 10.1186/s40478-020-01042-0 (PMC7643327; doi:10.1186/s40478-020-01042-0)
Supplement: Supplementary file 5 — Additional file 5: Figure S5. Regional differences in CSF influx and amyloid burden. [file 40478_2020_1042_MOESM5_ESM.pdf]

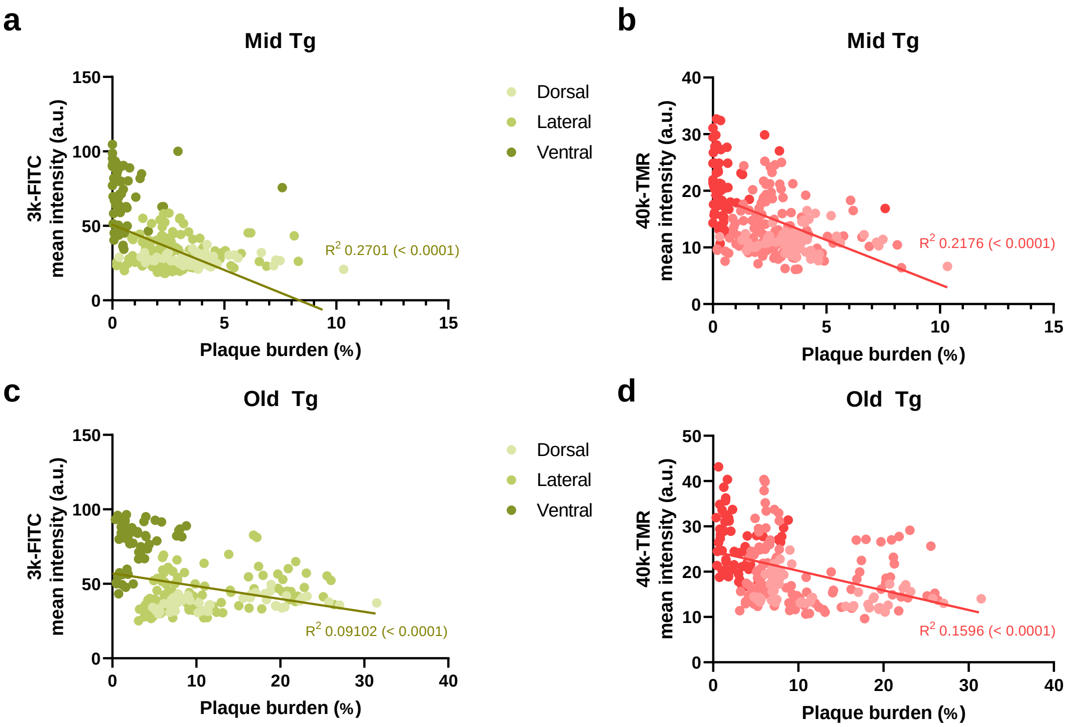

Additional file 5. Regional differences in CSF influx and amyloid burden

a-d. The relation between tracer signals and amyloid plaque burden across the brain cortex. The dorsal, lateral, and ventral regions are marked with different colors. The amyloid burden is higher in the order of ventral, lateral, and dorsal in both mid and old Tg mice. [(3 kDa; mid, $p < 0.0001$, $R^2 = 0.2701$; old, $p < 0.0001$, $R^2 = 0.09102$), (40 kDa; mid, $p < 0.0001$, $R^2 = 0.2176$; old, $p < 0.0001$, $R^2 = 0.1596$)] (n = 9, mid Tg, n = 8, old Tg, 7 slices/mouse).
